# Supplementary material for: COVID-19 cross-sectional study in Maricá, Brazil: The impact of vaccination coverage on viral incidence
Source: PLoS One. 2022 Sep 19;17(9):e0269011. doi: 10.1371/journal.pone.0269011 (PMC9484680; doi:10.1371/journal.pone.0269011)
Supplement: S1 Table — (PDF) [file pone.0269011.s001.pdf]

S1 Table. Detailed sociodemographic, non-pharmacological measures and clinical data from participants.

Characteristics	Participants											
	1st Cycle			2nd Cycle			3rd Cycle			Overall		
	Global	Ig+[a]	RT-PCR+	Global	Ig+	RT-PCR+	Global	Ig+	RT-PCR+	Global	Ig+	RT-PCR+
		%(no.)			%(no.)			%(no.)			%(no.)	
	100 (n=363)	100 (n=152)	100 (n=9)	100 (n=384)	100 (n=196)	100 (n=7)	100 (n=387)	100 (n=231)	100 (n=4)	100 (n=1134)	100 (n=579)	100 (n=20)
**Schooling profile**												
No schooling	2 (7)	3 (4)	11 (1)	2 (9)	3 (6)	14 (1)	2 (9)	1 (3)	0 (0)	2 (25)	2 (13)	10 (2)
Incomplete junior high	23 (82)	24 (37)	11 (1)	18 (69)	15 (30)	29 (2)	23 (89)	23 (54)	50 (2)	21 (240)	21 (121)	25 (5)
Complete junior high	4 (16)	6 (9)	0 (0)	8 (30)	10 (20)	14 (1)	8 (31)	10 (23)	0 (0)	7 (77)	9 (52)	5 (1)
Incomplete high school	10 (35)	8 (12)	22 (2)	9 (34)	10 (19)	0 (0)	9 (35)	7 (17)	0 (0)	9 (104)	8 (48)	10 (2)
Complete High school	34 (123)	36 (54)	33 (3)	33 (128)	33 (64)	29 (2)	32 (122)	29 (66)	25 (1)	33 (373)	32 (184)	30 (6)
Technician school	2 (9)	3 (5)	0 (0)	2 (8)	2 (4)	14 (1)	3 (13)	3 (8)	25 (1)	3 (30)	3 (17)	10 (2)
Under graduated	6 (23)	5 (7)	22 (2)	10 (38)	8 (15)	0 (0)	6 (23)	7 (16)	0 (0)	7 (84)	7 (38)	10 (2)
Graduated	17 (63)	14 (22)	0 (0)	15 (58)	17 (33)	0 (0)	15 (58)	17 (39)	0 (0)	16 (179)	16 (94)	0 (0)
Postgraduation and specialization	1 (5)	1 (2)	0 (0)	3 (10)	3 (5)	0 (0)	2 (7)	2 (5)	0 (0)	2 (22)	2 (12)	0 (0)
**Social distance compliance (SDC)**												
Did SDC	90 (327)	91 (138)	100 (9)	94 (360)	96 (189)	100 (7)	88 (342)	89 (206)	75 (3)	91 (1029)	92 (533)	95 (19)
Keep in SDC	83 (303)	87 (132)	89 (8)	91 (348)	94 (185)	100 (7)	82 (316)	84 (193)	75 (3)	85 (967)	88 (510)	90 (18)
**Hands washing frequency**												
< 3 times	25 (89)	25 (38)	22 (2)	34 (132)	33 (65)	14 (1)	44 (170)	42 (96)	25 (1)	35 (391)	34 (199)	20 (4)
> 4 times	75 (274)	75 (114)	78 (7)	66 (252)	67 (131)	86 (6)	56 (217)	58 (135)	75 (3)	65 (742)	66 (380)	80 (16)
**Alcohol gel use**												
Frequent	92 (334)	95 (145)	67 (6)	86 (331)	91 (178)	100 (7)	87 (337)	87 (200)	100 (4)	88 (1002)	90 (523)	85 (17)
Sometimes	6 (23)	4 (6)	33 (3)	13 (49)	9 (18)	0 (0)	10 (40)	11 (26)	0 (0)	10 (112)	9 (50)	15 (3)
Rarely	1 (5)	1 (1)	0 (0)	1 (4)	0 (0)	0 (0)	3 (10)	2 (5)	0 (0)	2 (19)	1 (6)	0 (0)

Never	<1 (1)	0 (0)	0 (0)	0 (0)	0 (0)	0 (0)	0 (0)	0 (0)	0 (0)	<1 (1)	0 (0)	0 (0)
**Mask use**												
Frequent	94 (340)	95 (145)	89 (8)	88 (338)	91 (179)	86 (6)	88 (342)	87 (202)	100 (4)	90 (1020)	91 (526)	90 (18)
Sometimes	5 (19)	3 (5)	11 (1)	11 (44)	9 (17)	14 (1)	11 (42)	12 (28)	0 (0)	9 (105)	~9 (50)	10 (2)
Rarely	1 (3)	1 (2)	0 (0)	<1 (1)	0 (0)	0 (0)	1 (3)	<1 (1)	0 (0)	<1 (7)	<1 (3)	0 (0)
Never	<1 (1)	0 (0)	0 (0)	<1 (1)	0 (0)	0 (0)	0 (0)	0 (0)	0 (0)	<1 (2)	0 (0)	0 (0)
	100 (n=86)	100 (n=33)	100 (n=6)	100 (n=105)	100 (n=51)	100 (n=5)	100 (n=98)	100 (n=53)	100 (n=2)	100 (n=289)	100 (n=137)	100 (n=13)
**Symptoms reported**												
Fever	13 (11)	18 (6)	33 (2)	19 (20)	18 (9)	60 (3)	10 (10)	11 (6)	0 (0)	14 (41)	15 (21)	38 (5)
Headache	42 (36)	42 (14)	50 (3)	39 (41)	41 (21)	60 (3)	34 (33)	42 (22)	0 (0)	38 (110)	42 (57)	46 (6)
Ageusia	9 (8)	9 (3)	33 (2)	6 (6)	12 (6)	40 (2)	8 (8)	11 (6)	0 (0)	8 (22)	11 (15)	31 (4)
Anosmia	12 (10)	9 (3)	33 (2)	6 (6)	12 (6)	40 (2)	7 (7)	9 (5)	0 (0)	8 (23)	10 (14)	31 (4)
Cough	47 (40)	39 (13)	67 (4)	39 (41)	45 (23)	60 (3)	38 (37)	49 (26)	50 (1)	41 (118)	45 (62)	62 (8)
Body ache	37 (32)	33 (11)	67 (4)	25 (26)	33 (17)	60 (3)	31 (30)	40 (21)	0 (0)	30 (88)	36 (49)	54 (7)
Throat ache	38 (33)	36 (12)	67 (4)	28 (29)	31 (16)	40 (2)	32 (31)	40 (21)	0 (0)	32 (93)	36 (49)	46 (6)
Dizziness	15 (13)	12 (4)	0 (0)	10 (10)	18 (9)	40 (2)	18 (18)	26 (14)	0 (0)	14 (41)	20 (27)	15 (2)
Running nose	62 (53)	58 (19)	50 (3)	58 (61)	61 (31)	40 (2)	58 (57)	66 (35)	50 (1)	59 (171)	62 (85)	46 (6)
Diarrhea	10 (9)	9 (3)	17 (1)	10 (11)	14 (7)	40 (2)	12 (12)	19 (10)	50 (1)	11 (32)	15 (20)	31 (4)
Vomit	7 (6)	6 (2)	17 (1)	2 (2)	2 (1)	0 (0)	9 (9)	13 (7)	50 (1)	6 (17)	7 (10)	8 (1)

[a]Participants CLIA IgM and/or IgG positives.
