# Supplementary material for: COVID-19 cross-sectional study in Maricá, Brazil: The impact of vaccination coverage on viral incidence
Source: PLoS One. 2022 Sep 19;17(9):e0269011. doi: 10.1371/journal.pone.0269011 (PMC9484680; doi:10.1371/journal.pone.0269011)
Supplement: Attachment — Submitted filename: A Tanuri PlosOne Rebbutal Letter 3.docx [file pone.0269011.s004.docx]

To PlosOne

REF: Rebuttal Letter Manuscript PONE-D-22-03333.

Rio de Janeiro, 5/3/2022

Dear Editor

Thank you for the excellent review done in our manuscript entitled “COVID-19 population-based survey in Maricá, Brazil: the impact of vaccination in viral incidence.” I am sure that reviewers´ comments will make the article clearer and more interesting for readers.

As advised by the Editor we have asked a native English speaker to review our manuscript and all corrections are in the new version.

Thank you for your attention and I hope our modifications satisfy the reviewers.

Sincerely Yours

Amilcar Tanuri

UFRJ
